# Substance Use Disorders and COVID-19: Multi-Faceted Problems Which Require Multi-Pronged Solutions

**DOI:** 10.3389/fpsyt.2020.00714

**Published:** 2020-07-21

**Authors:** Wossenseged Birhane Jemberie, Jennifer Stewart Williams, Malin Eriksson, Ann-Sofie Grönlund, Nawi Ng, Marcus Blom Nilsson, Mojgan Padyab, Kelsey Caroline Priest, Mikael Sandlund, Fredrik Snellman, Dennis McCarty, Lena M. Lundgren

**Affiliations:** ^1^ Department of Social Work, Umeå University, Umeå, Sweden; ^2^ Centre for Demography and Ageing Research (CEDAR), Umeå University, Umeå, Sweden; ^3^ The Swedish National Graduate School for Competitive Science on Ageing and Health (SWEAH), Department of Health Sciences, Faculty of Medicine, Lund University, Lund, Sweden; ^4^ Department of Epidemiology and Global Health, Faculty of Medicine, Umeå University, Umeå, Sweden; ^5^ Research Centre for Generational Health and Ageing, Faculty of Health, University of Newcastle, Callaghan, NSW, Australia; ^6^ School of Public Health and Community Medicine, Institute of Medicine, Sahlgrenska Academy, University of Gothenburg, Gothenburg, Sweden; ^7^ MD/PhD Program, School of Medicine, Oregon Health & Science University, Portland, OR, United States; ^8^ Psychiatry Unit, Department of Clinical Science, Umeå University, Umeå, Sweden; ^9^ Oregon Health & Science University- Portland State University, School of Public Health, Portland, OR, United States; ^10^ Cross-National Behavioral Health Laboratory, Graduate School of Social Work, University of Denver, Denver, CO, United States

**Keywords:** substance use disorder (SUD), COVID-19, addiction care, integrated care, social capital, pandemic, evidence-based policies and practices, risk environment

## Abstract

COVID-19 shocked health and economic systems leaving millions of people without employment and safety nets. The pandemic disproportionately affects people with substance use disorders (SUDs) due to the collision between SUDs and COVID-19. Comorbidities and risk environments for SUDs are likely risk factors for COVID-19. The pandemic, in turn, diminishes resources that people with SUD need for their recovery and well-being. This article presents an interdisciplinary and international perspective on how COVID-19 and the related systemic shock impact on individuals with SUDs directly and indirectly. We highlight a need to understand SUDs as biopsychosocial disorders and use evidence-based policies to destigmatize SUDs. We recommend a suite of multi-sectorial actions and strategies to strengthen, modernize and complement addiction care systems which will become resilient and responsive to future systemic shocks similar to the COVID-19 pandemic.

## Introduction

Persistent use of psychoactive substances increases risk of substance use disorders (SUDs) – biopsychosocial disorders with multiple risk factors interacting at individual and contextual levels resulting in co-morbid health conditions and affecting people from all social and economic backgrounds (1, 2). The health consequences of SUDs (e.g., cardiovascular diseases, respiratory diseases, type-2 diabetes, immune and central nervous system depression, and psychiatric disorders) and the associated environmental challenges (e.g., housing instability, unemployment, and criminal justice involvement) increase risk for COVID-19 (3–7). COVID-19 adds to the complexity of SUD as it affects the lives of individuals with SUD.

## The Intersection of Substance Use Disorder and COVID-19

SUDs and COVID-19 intersect on five dimensions. First, drug and alcohol use are often communal (e.g., sharing blunts, smoking pipes, or syringes) and may contribute to the spread of COVID-19 (8). Second, many individuals with SUD have limited financial resources, unstable housing and limited access to clean water and soap increasing their risk of infection (8, 9). Third, co-morbidities prevalent among people with SUD are associated with more severe COVID-19 symptoms, complications and fatalities and increase vulnerability to COVID-19 (3–7). Fourth, COVID-19 public health mitigation measures (i.e., physical distancing, quarantine and isolation) may exacerbate loneliness, mental health symptoms, withdrawal symptoms and psychological trauma (10–13). Fifth, COVID-19 mitigation measures are likely to inhibit access to SUD treatment services (8). For many patients, the face-to-face interaction with practitioners is a key therapeutic ingredient for their recovery. These collisions between COVID-19 and SUD lead to more severe outcomes, especially among older adults with SUD who already have limited individual and social resources (3).

Finally, because COVID-19 burdens health care and social services, resources may be diverted from addiction services at a time when people with SUD need additional interventions. Lived experience of stigma and discrimination may also deter people with SUD from seeking healthcare during the pandemic (14). It is important that addiction care and social service providers are made aware regarding the vulnerability of the different sub-populations to COVID-19. This will enable providers to treat people with SUD in a non-stigmatizing and nondiscriminatory manner and provide appropriate services (15–17).

The COVID-19 pandemic has serious implications for individuals with SUD including long-term socioeconomic and public health effects. Drawing on evidence from previous economic and health disasters, we examine the potential economic, public health and social implications of COVID-19 and SUDs, and provide a short description of efforts to ensure continuity of addiction services during the pandemic. The article closes with recommended policy approaches and solutions for tackling SUD within both the context of COVID-19 and the resulting shock to health and economic systems.

## COVID-19 Induced Economic, Public Health, and Social Challenges

### Unemployment, Substance Use, and Mental Health Comorbidity

The COVID-19 pandemic impacted the global economy leaving millions of people unemployed, without a social safety net and limited access to healthcare and social services (18, 19). The associations of involuntary or unexpected unemployment with SUD and mental health, and the positive effect of reemployment are well established. When individuals with SUD lose the structure of employment and sense of purpose, substance use and SUD symptom severity may increase (9, 17, 20–30). Home foreclosure in the United States (US) was associated with a delayed onset of depression and anxiety after controlling for pre-existing depression and anxiety (31). As pandemic-related unemployment soars, and home foreclosures and housing eviction rises, there may be increases in mental health and SUD problems.

Studies of economic crises, similar to the pandemic-induced recession, suggest that SUD-related mortality and suicide will increase. Unemployment in Sweden during the severe recession in the 1990s was associated with alcohol-attributable hospitalization and mortality (32) and suicide during a 12-year follow-up (33). An analysis of economic changes in 26 European Union (EU) countries over three decades showed that increases in unemployment were associated with a 28% increase in mortality from SUD and a 4.5% increase in suicide (34). During the 2008–2010 financial crisis socioeconomic vulnerability among millennials (compared to older generations) was associated with increased alcohol and drug use disorders in the US (35).

### Cuts in Public Expenditures on Healthcare and Social Care: “Where Recession Hurts, Austerity Kills”

Cuts in healthcare and social care expenditures, measures taken in response to the economic impact of COVID-19, may exacerbate the public health effects of acute economic change (20, 36–39). These changes, compounded with unemployment and loss of income in the post-COVID-19 period, may affect resource allocation and priority setting, widen socioeconomic disparities, and magnify the marginalization of individuals with SUDs (40, 41).

When an economic crisis worsens and austerity measures are implemented, public health infrastructure can be stressed and the “risk environments” for SUD may expand (42). Poverty drives people to rely on informal economies (e.g., sex work, drug dealing) associated with illicit drug use. Compounded by weakened public health infrastructure, this can lead to a rise in preventable infectious diseases. The rapid increase in the HIV infection rate among persons who inject drugs (PWIDs) after the collapse of the Soviet Union and the formation of newly independent states in Eastern Europe, reflected the dismantling of public health infrastructures and increased unemployment (43). Similarly, the 2008–2010 financial crisis in Greece resulted in ongoing economic depression. Severe austerity measures led to a 40% reduction in hospital budgets by 2013 (44). However, the austerity measures also resulted in a 30% increase in the utilization of public healthcare services (44). Further, one-month prevalence of major depression increased from about 3% in 2008 to 8% in 2011 (45) and suicide mortality increased 56% between 2007 and 2011 (46, 47). The austerity also led to budget cuts for harm reduction and opioid treatment programs. Between 2008 and 2010 the number of people who used drugs increased 12% and was much higher for adults between 35 and 64 years (88%) most likely due to relapse (48). Finally, the number of HIV infected people among PWIDs in Greece increased 16-fold between 2010 (n = 15 cases) and 2011 (n = 260 cases) (49).

The ongoing pandemic is straining healthcare systems across the globe. Data from the Swedish Perioperative Register (SPOR) reflect a 74% decline in elective surgeries in April 2020 compared to April 2019 due to acute reorganization of healthcare to respond to COVID-19 (50). If governments react to the economic crisis through reductions in spending for healthcare and social care, the stress on healthcare may be exacerbated and lead to a resource triage and decline in healthcare quality (51).

People with SUD may be further affected as the COVID-19 impact worsens. This group already faces stigma and discrimination from the general public (52), policy makers (53, 54) and healthcare workers (14, 55–58). Resource allocation and clinical practice with embedded stigma and discrimination has a prohibitive effect on healthcare utilization by individuals with SUD (14). Therefore, a reasonable, open and transparent, inclusive, accountable, and responsive process is necessary in priority setting and resource allocation during and after COVID-19.

### Changes in Drug Use Patterns During the COVID-19 Induced Systemic Shock

Confinement rules, unemployment and fiscal austerity measures during and following the pandemic period can affect the illicit drug market and drug use patterns. European Monitoring Centre for Drugs and Drug Addiction (EMCDDA) and Europol analyses and data from the Global Drug Survey (GDS) suggest that there has been a shift in drug market and drug use patterns during the pandemic (59, 60). While the use of several psychoactive substances increased, use of recreational synthetic drugs, such as MDMA, diminished likely due to closure of clubs and festival avenues in several European countries.

Economic crises in the United States between 1959 and 2003 were associated with increased adolescent cannabis and illicit drug use, and elevated involvement in illicit drug markets (61). As people who use drugs lose income and can no longer afford their primary drug of use, suppliers may adulterate drugs or introduce novel psychoactive substances with unknown risks for overdosing and infectious disease transmission. A Hungarian study reported a shift from heroin and amphetamine injection to synthetic cathinone (bath salt) and reduced availability of heroin after the 2008–2010 financial crisis (62). Synthetic cannabinoids (spice), similarly, became a primary drug of use among the homeless population following a ban on novel psychoactive substances in the United Kingdom (63). Finally, a wastewater analysis from Northern Italy in 2009 noted a reduction in metabolites from expensive drugs (e.g., cocaine and heroin) and increased metabolites from less expensive drugs (e.g., methamphetamine and cannabis) (64).

### Bereavement and Loneliness: Lasting Effects of the COVID-19 Pandemic

In addition to the economic peril in the post-COVID-19 period, the pandemic is traumatizing people. Shrinking social networks and deaths from COVID-19 leaves many without coping resources (65). Social isolation, loneliness, death of loved ones, complicated grief, and prolonged bereavement are associated with problematic substance use and relapse both in younger and older adults, and can adversely affect mental health (17, 66–75).

Older adults who are living alone are more likely to have SUD when compared to married older adults (5). Living alone is also associated with depression in older adults (76). The current pandemic potentially adds to the already high percentages of older adults living alone (77). For some older adults with depression, the pandemic-related bereavement might also affect their remission (78). Unless socially protective measures are taken, the post-pandemic period will likely exacerbate these risk factors for substance use and mental health disorders.

## Current Addiction Care Practice During the COVID-19 Pandemic

Countries differ in legal and regulatory frameworks and the organization of addiction care systems; addiction treatment, however, is recognized internationally as an essential service that should be maintained even in a disaster or pandemic (79). Many countries have national policies guiding the implementation and application of interventions linked to health and social care systems. During the pandemic, psychiatric and addiction care services are making efforts to ensure continuity of care while mitigating the risk for spreading COVID-19 infections (80, 81). In Sweden, the National Board of Health and Welfare posted informational materials on how to prevent the risk of COVID-19 transmission in opioid treatment programs (OTPs); in the United States, the Substance Abuse and Mental Health Services Administration released guidance to allow safer administration of methadone during the pandemic. Most of the measures focus on reducing the number of outpatient treatment visits, increasing the use of telehealth and expanding take-home medication for OTPs (82). While these current actions mitigate the negative impact of COVID-19 on individuals with SUD, there remains a need to adopt proactive policies which support individuals with SUD and strengthen addiction care services.

## Policies and Strategies to Prevent and Treat SUD in the COVID-19 Context

SUD is a biopsychosocial disorder with multiple individual risk factors and consequences. SUD and mental health disorders also have distal determinants. Hence, interventions must be multipronged with community involvement and empowerment. It is important to adopt coordinated multi-sector strategies and innovative holistic approaches to benefit individuals with SUD.

### Protective Social Policies Can Improve Living Conditions and Access for Addiction Care Services

Social policies impact health, directly and indirectly, through proximal and distal social determinants such as income, housing, employment, education, place of residence and social capital. Outcomes measured at the population level, mask effects on vulnerable groups and individuals with substance use disorders (83). Program evaluations do not always account for unintended consequences although realist evaluation methods take a different approach in seeking to answer what works, for whom, in what respects, to what extent, in what contexts and how.

Strong financial assistance systems can alleviate the negative impact of economic peril on mental health, during COVID-19 pandemic induced recession (22, 41, 84). A study of 26 European countries, with cause-specific mortalities as the outcomes (1970–2007) found that countries with stronger social protection (employment support and welfare systems) fared better compared to their counterparts (34). In a Norwegian study, reemployed individuals were 65% less likely to become harmful alcohol users compared with those who stayed unemployed (85). These studies suggest that public expenditures for labor market programs supporting gainful employment or earning capacity were associated with reductions in alcohol-related mortality and suicide.

Strong public safety nets for health, unemployment and social care insurances, support vulnerable groups such as people with mental health disorders and SUD, and ensure that they have access to treatment despite loss of income or employment related health insurance (63, 86). The number of individuals receiving care for opioid use disorder, for example, increased nearly twofold after Oregon’s Medicaid expansion in 2014 (87). Given the acute reorganization of healthcare during the pandemic and decrease in healthcare utilization, healthcare plans and resources can be redirected to making structural changes to reduce health disparities and promote health in vulnerable populations (88).

### Develop and Expand Integrated Primary Care, Addiction, and Mental Health Care Systems

National and local policymakers need to accept that substance use disorders, as any other biopsychosocial disorder (e.g., diabetes), often require several intervention components and multiple treatment episodes. These include services for alcohol and drug, mental health and medical problems plus linkages to unemployment services, housing services, and family support services. In many societies, there is little understanding of the complexities of SUD. Many countries have regressive and punitive national policies which are based on prohibitive and moralistic views rather than evidence-based policies promoting the integration of biopsychosocial services and care for individuals with SUD. The lack of willingness to give up on the legacy of separate health, addiction and mental health care systems, true for many countries, further reduces the likelihood that clients with SUD (who as a result of COVID may have developed a number of co-occurring disorders) will receive integrated care, especially in limited resource settings. Parallel treatment between several care providers means that the patient is responsible for the coordination of treatment between different agencies. An integrated care system, however, reduces this burden and can address coexisting conditions simultaneously (89). Compared to fragmented care, integrated care can increase access to healthcare for individuals with SUD, and may reduce infectious diseases such as COVID-19.

### Implement Professional Education About SUD and Co-Occurring Disorders

Health professionals face challenges while using empirically supported screening, assessment, referral treatment, and follow-up for SUD and co-occurring disorders because they lack training about causes and consequences of substance use (including the biomedical aspects), and have limited training with evidence based practices (90, 91). In the United States, medical, nursing, and social work programs are beginning to add SUD curricula to their training (92). Given the likely effects of COVID-19 and other diseases on SUD populations, it is even more critical that physician, nursing, psychology, and social work education programs include addiction and SUD content in their core-curriculum. Rapid training of addiction care professionals, in an emergency situation, (e.g., the current COVID-19 crisis) can help to control rapid outbreaks and provide safe addiction care.

### Integrate IT Solutions to Strengthen and Modernize the Addiction Care System

As the current pandemic and the economic crisis threatens health and social care expenditures, information and communication technologies can play vital roles in improving healthcare and social services. New technology solutions that can modernize and strengthen the health and social care systems should be studied, and evaluated for cost-effectiveness.

The Internet of Things has shown effectiveness in monitoring elderly health and medication adherence (93–96). OTPs and other medical treatments for individuals with SUD may benefit from similar technology. Individuals with SUD can learn to manage their substance use and self-monitor symptoms. This can lead to reduced outpatient treatment visits and hospitalizations.

Telehealth has been used in some settings during the COVID-19 pandemic to maintain access to treatment (97). A systematic review and meta-analysis reported that telehealth, especially live video interaction with therapists, had significant positive effects on patient mental health (98, 99). A non-randomized trial found that telehealth-delivered treatment for opioid use disorder was associated with better one-year retention compared to in-person delivered treatment (100). Studies have showed that older adults can benefit from telehealth services through reduced visits to emergency departments, increased knowledge of infectious diseases prevention, and improved social functioning and mental health (101, 102). Future studies should investigate how the telehealth services provided during COVID-19, impacted SUD treatment outcomes and stigma.

Concerns related to telehealth services, in addition to scarcity of evidence on their effectiveness, focus on their accessibility (103). Limited access to smartphones and internet services leaves millions of people without access to those services (104). People with SUD may not afford such devices and might not have access to telehealth. One possible solution for this disparity can be mobile health (m-health) technologies. These are less costly and are effective for SUD treatment (105); they might also be utilized for pandemic surveillance in vulnerable groups (106, 107). Social policies focusing on equitable resource allocation and social support (such as health insurance and income insurance) can also address this disparity.

Artificial intelligence (AI), another promising technology that could be used during emergency situations, could support trained clinicians to make treatment decisions. Currently, the research on the potential use and benefits of AI in addiction care and mental health services is in early development and needs to address important scientific, legal and ethical issues (108, 109). Current AI research is focused on assisting addiction care practitioners with treatment for alcohol use disorder (110), identifying and preventing relapse (111), and identifying risk factors (112, 113). Practitioners should, however, be aware that algorithms can be subject to biases (due to misclassification and measurement error, missing data, and small sample size) (108). The implication of such biases can be severe as they might create disparities in addiction care (108, 109). Involving addiction care specialists and patient advocacy groups from the beginning in the development of AI can facilitate innovative, ethical, acceptable, and effective solutions.

Finally, when the technology around unmanned aerial vehicles (drones) improves and becomes cost-effective and ethical and legal issues are addressed, harm reduction kits, and medications could be delivered to individuals with SUD (114–116). Drones can deliver medications (e.g., naloxone) and save lives especially in highly congested cities and rural areas. They can also be used as an alternative for take-home medication for OTPs. Drones are already used for medical delivery services in emergency situations (115). However, current policies and views on harm reduction and addiction vary from country to country, and this might influence the acceptability of drones as kit-delivery vehicles.

### Mobilization of Community Social Capital

During the COVID-19 pandemic voluntary efforts from community members and non-governmental organizations seek to help vulnerable groups. Mental health hotlines opened so that older adults can talk to professionals if they feel lonely or worried. Mobile apps and chat groups are now available for digital support. Community level coalitions and inclusion will be needed to support individuals with substance use and mental health disorders.

Mobilization of community social capital is an important resource in disaster management (117). A socially cohesive community with strong networks of civic engagement and norms of reciprocity and trust (118) may be better able to prepare for, manage, and recover from systemic shocks such as the COVID-19 pandemic (119). Resources (such as social support) from strong community networks, however, often require adhering to the dominant norms in a particular community. Thus, the same mechanisms that provide support based on reciprocity norms, might lead to increased social exclusion of outsiders who do not conform to the dominant norms (120, 121). For this reason, the focus should be on policies which promote parity for the treatment of substance use disorder to that of other biopsychosocial health conditions, support the development and implementation of community initiatives that complement addiction and mental health care services and can be leveraged during disaster (14, 54).

### Strengthening of Cross-National Collaboration

Many illicit substances and their precursors are manufactured and transported through multiple countries, before reaching users. Collaboration between countries can counteract the interplay between SUD and economic crises. After the 2010–2011 HIV outbreak among PWID in Greece, the European Monitoring Centre for Drugs and Drug Addiction (EMCDDA) and the European Centre for Disease Prevention and Control (ECDC) were instrumental in setting priorities for responding to and controlling the rapid HIV infection rate (122). EMCDDA also provides EU countries with early warning systems for novel psychoactive substances and new drug patterns which can emerge during economic crises.

The World Health Organization and the United Nations Office on Drugs and Crime are international organizations guiding efforts to develop and expand effective, evidence-based and ethical treatment for substance use disorders (79). Hence, national governments should continue funding these organizations, especially during COVID-19 and similar disease outbreaks. Strengthening community treatment capacity is essential during disaster and public health emergencies.

## Conclusion

As globalization continues, COVID-19 is unlikely to be the last pandemic, and there will undoubtedly be subsequent global economic crises. These crises, compounded by austerity measures, will disproportionately burden people with SUD due to accumulated social, economic, and health inequities.

Ad hoc measures taken to ensure continuity of care might alleviate some of the challenges these groups face in emergency situations. Evidence-based, collective, and proactive policies and actions are necessary to strengthen and modernize addiction and mental health services.

The acknowledgement of SUD as a biopsychosocial condition and its destigmatization by policy makers and practitioners are essential components for comprehensive multi-sectorial strategies which will protect and address the needs of people with SUD.

COVID-19 presents opportunities to: adopt social protective policies; shift from fragmented health and addiction care systems to integrated care systems; mobilize community social capital; train healthcare and social care professionals on SUD and mental health disorder, and identify and integrate evidence-based information technology and digital tools into addiction care systems. Only then, will it be possible to provide equitable health and social care to people with SUDs and to have addiction care services which are resilient in the face of future systemic shocks.

## Data Availability Statement

The original contributions presented in the study are included in the article/supplementary material; further inquiries can be directed to the corresponding author.

## Ethics Statement

Ethical approval was not needed for this article as no animal nor human studies are presented, and there are no potentially identifiable human images or data.

## Author Contributions

Writing—original draft: WJ, ME, MP, KP, DM, LL, Writing—Review and editing: WJ, JS, ME, A-SG, NN, MB, MP, KP, MS, FS, DM, LL. Conceptualization: WJ, JS, NN, DM, LL. Investigation: WJ. Formal Analysis: WJ. Funding acquisition: LL. All authors contributed to the article and approved the submitted version.

## Funding

Grants from The Swedish Research Council for Health, Working Life and Welfare (FORTE) *Grant no. 2016-07213* and *Grant No. 2019-01453* have supported this study. An award from the National Institute on Drug Abuse (*F30 DA044700*) supported KP’s participation in the development of the manuscript. The funding organizations were not involved in the design of the study, data collection, data analysis, the interpretation of data, or writing of the manuscript.

## Conflict of Interest

The authors declare that the research was conducted in the absence of any commercial or financial relationships that could be construed as a potential conflict of interest.
